# Hypoxic condition enhances chondrogenesis in synovium-derived mesenchymal stem cells

**DOI:** 10.1186/s40824-018-0134-x

**Published:** 2018-09-26

**Authors:** Hyun Cheol Bae, Hee Jung Park, Sun Young Wang, Ha Ru Yang, Myung Chul Lee, Hyuk-Soo Han

**Affiliations:** Department of Orthopedic Surgery, Seoul National University Hospital, Yongondong Chongnogu, Seoul, 110-744 Republic of Korea

**Keywords:** Synovium-derived mesenchymal stem cells (SDSCs), Hypoxia, Chondrogensis, Oxygen

## Abstract

**Background:**

The chondrogenic differentiation of mesenchymal stem cells (MSCs) is regulated by many factors, including oxygen tensions, growth factors, and cytokines. Evidences have suggested that low oxygen tension seems to be an important regulatory factor in the proliferation and chondrogenic differentiation in various MSCs. Recent studies report that synovium-derived mesenchymal stem cells (SDSCs) are a potential source of stem cells for the repair of articular cartilage defects. But, the effect of low oxygen tension on the proliferation and chondrogenic differentiation in SDSCs has not characterized. In this study, we investigated the effects of hypoxia on proliferation and chondrogenesis in SDSCs.

**Method:**

SDSCs were isolated from patients with osteoarthritis at total knee replacement. To determine the effect of oxygen tension on proliferation and colony-forming characteristics of SDSCs, A colony-forming unit (CFU) assay and cell counting-based proliferation assay were performed under normoxic (21% oxygen) or hypoxic (5% oxygen). For in vitro chondrogenic differentiation, SDSCs were concentrated to form pellets and subjected to conditions appropriate for chondrogenic differentiation under normoxia and hypoxia, followed by the analysis for the expression of genes and proteins of chondrogenesis. qRT-PCR, histological assay, and glycosoaminoglycan assays were determined to assess chondrogenesis.

**Results:**

Low oxygen condition significantly increased proliferation and colony-forming characteristics of SDSCs compared to that of SDSCs under normoxic culture. Similar pellet size and weight were found for chondrogensis period under hypoxia and normoxia condition. The mRNA expression of types II collagen, aggrecan, and the transcription factor SOX9 was increased under hypoxia condition. Histological sections stained with Safranin-O demonstrated that hypoxic conditions had increased proteoglycan synthesis. Immunohistochemistry for types II collagen demonstrated that hypoxic culture of SDSCs increased type II collagen expression. In addition, GAG deposition was significantly higher in hypoxia compared with normoxia at 21 days of differentiation.

**Conclusion:**

These findings show that hypoxia condition has an important role in regulating the synthesis ECM matrix by SDSCs as they undergo chondrogenesis. This has important implications for cartilage tissue engineering applications of SDSCs.

## Background

Mesenchymal stem cells (MSCs) have been promising sources for cell-based regenerative therapy for articular cartilage defects [1, 2]. Clinical and pre-clinical studies have shown variable outcomes following MSC transplantation for treatment of focal chondral and osteochondral defects. Synovium-derived mesenchymal stem cells (SDSCs) have superior expansion ability and chondrogenic potential than MSCs from other sources [3, 4]. Although the reparative potential of SDSCs and bone marrow-derived MSCs (BM-MSCs) is similar, an in vivo chondrogenic assay demonstrated that SDSCs produce more cartilage matrix than BM-MSCs [3, 4]. BM-MSCs preferentially differentiate into bone, while SDSCs differentiate better into chondrocytes [5]. Furthermore, SDSCs can be obtained from patients by minimally invasive techniques, which could allow their use as a source of cells for cartilage regeneration.

The chondrogenic differentiation of MSCs is controlled by many factors, such as growth factors, cytokines and 3-dimensional scaffolds [6–8]. In recent years, it has been demonstrated that oxygen levels are important in the control of MSC proliferation and can also drive MSC differentiation [9–11]. Hypoxia, a condition of low oxygen supply, has already been shown by others to stimulate chondrogenic matrix production in chondrocytes and MSCs. Low oxygen tensions enhanced expansion potential of MSC including human umbilical cord blood -derived MSC, and adipose derived MSC [11, 12].

In addition, articular cartilage is avascular and exists at a low oxygen tension of (1 to 7%). The oxygen tension inside it ranges from 1% near subchondral bone to 7% close to joint surface [13, 14]. Consequently, hypoxia is found to be indispensable in cartilage physiology. In human articular cartilage, hypoxia increased expression level of sex determining region Y-box 9(SOX9), which was the essential transcription factor in chondrogenesis [15]. In chondrocyte culture systems it has been shown that under hypoxia there is increased synthesis of extracellular matrix by chondrocytes, and this has been extended to stem cells from bone marrow and adipose tissue undergoing chondrogenesis [16, 17].

Thus, oxygen tension seems to be an important regulatory factor in the proliferation, differentiation and matrix production of chondrocytes. But, the effect of low oxygen tension on the chondrogenic differentiation in SDSCs has not characterized. In this study, we investigated the effects of hypoxia on chondrogenesis in SDSCs.

## Methods

### Primary human synovium-derived mesenchymal stem cells (SDSCs) culture

In this experimental study, synovium tissues were obtained from five female osteoarthritis patients (age 66 to 72 years) undergoing total knee arthroplasty (TKA). In all patients, the Kellgren Lawrence grade was 4 and osteoarthritis had progressed at the medial side of knee. Synovium was harvested from the suprapatellar pouch. Ethical approval for this study was obtained from Seoul National University Hosiptal Institutional Review Board. Those who had inflammatory arthritis, prior knee joint infection, and intraarticular trauma were excluded. Synovial tissue was minced in phosphate-buffered saline (PBS) and digested with 0.02% collagenase (Sigma, St. Louis, Missouri) overnight. Cells were filtered from undigested tissue with 70 μm sieves and centrifuged at 1,500 rpm for 5 min. Then, cells were cultured in low glucose Dulbecco’s modified Eagle’s medium (LG-DMEM, Gibco, UK) with 10% fetal bovine serum (FBS) and 1% penicillin/ streptomycin/amphotericin at 37 °C with 5% CO2. The medium was changed after 48 h and nonadherent cells were removed during this procedure. In our previous study, we investigated changes in the proliferative capacities, chondrogenic phenotypes, and gene expression profiles of SDSCs at passage 0, 1, 2, 4, 6, and 8 [35]. The results demonstrated that genetic and phenotypic changes occur between passage 2 and 4 and that late passage cells differentiate less well to chondrocytes than early passage cells. Thus, we used early passage SDSCs (P2) in this study.

### Proliferation and colony-forming unit assay

To determine the effect of oxygen tension on proliferation of SDSCs, SDSCs were plated in triplicate at 1 × 10^5^ SDSCs per 100 mm diameter sterile dish (Becton Dickinson Canada Inc.) and cultured under normoxia (21% O_2_) or hypoxia (5% O_2_). To avoid an over-growth in culture plate, total cells were sub-cultured twice each week for 21 days. Total cell counts of trypsinized SDSCs under either normoxic (21% O_2_) or hypoxic (5% O_2_) conditions were calculated by using trypan blue staining and hemocytometer counting of small aliquots of SDSCs s in expansion medium.

To determine the effect of oxygen tension on colony-forming characteristics of SDSCs, SDSCs were plated in triplicate at 1 × 10^5^ SDSCs per 100 mm diameter sterile dish (Becton Dickinson Canada Inc.) and cultured under normoxia (21% O_2_) or hypoxia (5% O_2_). After the first week, the non-adherent cell population was removed by aspiration and culture media were replenished twice each week. After the CFU-F culture period finished, the dishes were fixed with 10% buffered formalin (3.8% formaldehyde), washed by using phosphate-buffered saline (PBS) (Life Technologies), and stained with 0.25% crystal violet solution (Sigma-Aldrich).

### Chondrogenesis of MSCs

5 × 10^5^ SDSCs were centrifuged at 1,500 rpm for 5 min to obtain cell pellets. Cell pellets were cultured in chondrogenic medium (LG-DMEM) containing 0.1 mmol/L ascorbic acid 2-phosphate, 100 nmol dexamethasone, 40 g/mL proline, 100 U/mL penicillin, 100 g/mL streptomycin, and ITS Premix (BD Biosciences, Massachusetts) supplemented with transforming growth factor beta 1 (TGF-ß1). SDSCs pellets were allowed to differentiate up to 21 days under either normoxic (21% O_2_) or hypoxic (5% O_2_) conditions. Medium was refreshed every 3–4 days.

### Quantitative real-time PCR analysis

Total RNA was extracted using TRIzol kit (Invitrogen, CA). RNA was reverse transcribed in a final volume of 20 mL using 0.5 mg of oligo dT and 200 U Superscript III RT (Invitrogen) for 30 min at 50 °C, followed by 2 min at 94 °C to inactivate the reverse transcriptase. Real-time PCR amplification was carried out in a total volume of 25 μL containing 6.25 μL water, 1.25 μL primer (9 mM), and probe (2.5 mM) and 12.5 μL TaqMan PCR 2X master mixture (Perkin-Elmer Applied Biosystems), 5 μL complementary DNA. The PCR conditions were as follows: after the initial activation of uracyl-N-glycosylase at 50 °C for 2 min, AmpliTaq Gold was activated at 95 °C for 10 min; the subsequent PCR condition consisted of 45 cycles of denaturation at 95 °C for 15 s and annealing extension at 60 °C for 1 min per cycle. During the PCR amplification procedure, the amplified products were continuously measured by determination of the fluorescence emission. The levels of the target gene expression were analyzed using the 2-ΔΔCt method [33] and they were normalized to a human GAPDH endogenous control (VIC/MGB Probe, Primer Limited; Perkin-Elmer Applied Biosystems), and the levels were presented as the relative expression. The PCR primer and probe sets for *COL2A1* (GGCGACGGCCCCCACGCCCACTCGC), *COL10A1* (ACTGCAAGGAGAGCCAGGGTTGCCA), *ACAN* (GATGGAGGGTGAGGTCTTTTACG), and *SOX9* (CCTCGGGAAGCTCTGGAGACTGCTG) were designed using the Primer Express software (Perkin-Elmer Applied Biosystems).

### Histology and immunohistochemistry

For histological evaluation of glycosaminoglycan (GAG) synthesis, cell pellets from each group were stained with Safranin-O and fast green staining at days 21. Staining was performed as described in our previous study [18]. To evaluate the production of type II and X collagen histologically, immunohistochemical staining was performed in each group at days 21 using mouse anti-human monoclonal antibodies for type II and X collagen (Neomarkers, California). Staining of type II and X collagen was examined separately and detail procedures were performed as described previously in our study [18].

### Western blot assay

The total cell lysates were isolated by sonication and the supernatant proteins (10 mg/lane) were subjected to Tris-Glycine gel (Invitrogen) electrophoresis, and transferred to a nitrocellulose membrane (Hybond, Amersham, Pisactaway, NJ). The membranes were blocked with 5% non-fat dry milk, and incubated with the mouse anti- type II and X collagen antibody in 1:3,000 dilution or anti-β-actin antibody (Sigma, St. Louis, MO) in 1:2,000 dilution. The blots were then reacted with a horseradish peroxidase conjugated anti-mouse secondary antibody (Pierce, Rockford, IL). The immunoreactive proteins were then visualized using ECL detection reagents (Supersignal West Dura, Pierce). Quantitative densitometrical analyses of Western blot images were performed using TINA software (Raytest, Isotopenmebgerate, Germany).

### Determination of the glycosaminoglycans content

The dimethylmethylene blue (DMB) method was used for the detection of the total glycosaminoglycan (GAG) contents in the pellet. At day 21 after cultivation in hypoxia or normoxia condition, the pellets were digested overnight at 60 °C in 20 μl of 10 U/ml papain (Sigma), 0.1 M sodium acetate, 2.4 mM EDTA, 5 mM l-cysteine pH 5.8. After centrifugation, 50-mL aliquots of the papain-digested extracts were pipetted into each well of 96-well plates and 250 mL of the DMB dye solution was added. The absorbance of these extracts was determined using an ELISA reader at 530 and 590 nm, and were compared to the absorbance of standard chondroitin-6-sulfate. The total GAG quantities per sample (μg/μg) were then normalized by their DNA contents as determined using PicoGreen (Invitrogen).

### Statistical analysis

Statistical differences between two groups were analyzed using the Student t test or one-way ANOVA. Statistical significance was set to *p* < 0.05 and was indicated with an asterisk (*) sign. Statistical non-significance was set to *p* > 0.05 and was indicated with an N.S (non-significance) sign. Results are presented as mean ± SD.

## Result

### Hypoxia stimulates proliferation and colony-forming characteristics of SDSCs

**Fig. 1:**
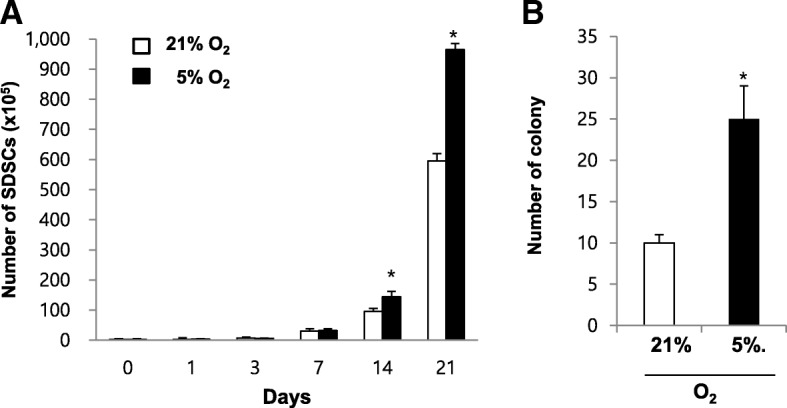
Hypoxia stimulates proliferation and colony-forming characteristics of SDSCs. The SDSCs were cultured for 21 days under normoxic (21% oxygen) or hypoxic (5% oxygen) conditions. (**a**)Total cell counts of were calculated by using trypan blue staining. (**b**) CFU-F assay was performed to determine the effect of oxygen tension on colony-forming characteristics of SDSCs

A proliferation assay was performed to determine the effect of oxygen tension on the proliferation of SDSCs. After the SDSCs at passage 2 were cultured for 21 days under normoxic (21% O_2_) or hypoxic (5% O_2_) conditions. Total cell counts were calculated by using trypan blue staining. A proliferation assay revealed that hypoxic conditions significantly increased proliferation of SDSCs on days 14, and 21 compared to that of SDSCs under normoxic culture (*p* < 0.05) (Fig. 1a). A CFU-F assay was performed to determine the effect of oxygen tension on colony-forming characteristics of SDSCs. At 18 days after cultivation, SDSCs cultured under hypoxic conditions had 2.5-fold higher colony number than those cultured under normoxic conditions (Fig. 1b).

### Chondrogenic culture of SDSCs and the effect of low oxygen tension on chondrogenic differentiation

**Fig. 2:**
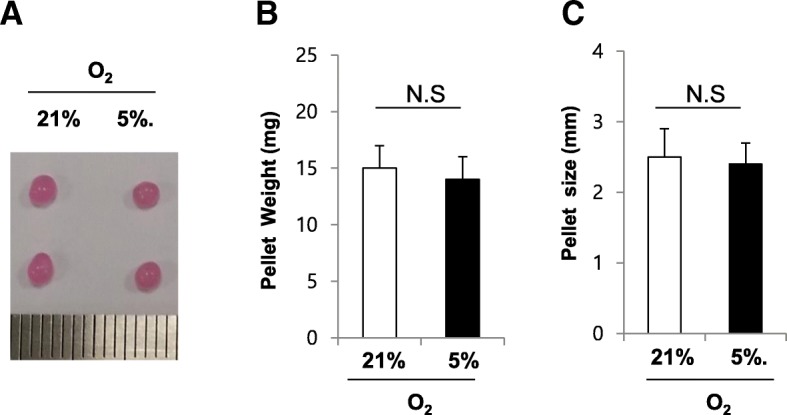
Chondrogenic culture of SDSCs and the effect of low oxygen tension on chondrogenic differentiation. SDSCs were concentrated to form pellets and Pellets of SDSCs were chondrogenic differentiated. 21 days after cultivation under normoxia and hypoxia condition, (**a**) Morphology of SDSC pellet. (**b**) Size of SDSC pellet. (**c**) weight of SDSC pellet

SDSCs were concentrated to form pellets and pellets of SDSCs were differentiated into the chondrogenic lineage for up to 21 days in the presence of TGF-β in either normoxic or hypoxic conditions. At 21 days after cultivation under normoxia and hypoxia condition, similar pellet gross were found for chondrogensis period under hypoxia and normoxia condition (Fig. 2a). Pellet cultured under hypoxic conditions at 21 days had slightly smaller pellet weight than those cultured under normoxic conditions. However, mean values were not statistically different (*p* = 0.73: Fig. 2b). Also, no difference was noted in pellet size between hypoxia and normoxia condition (Fig. 2c).

### Hypoxia induces chondrogenesis-related gene expression in SDSCs

**Fig. 3:**
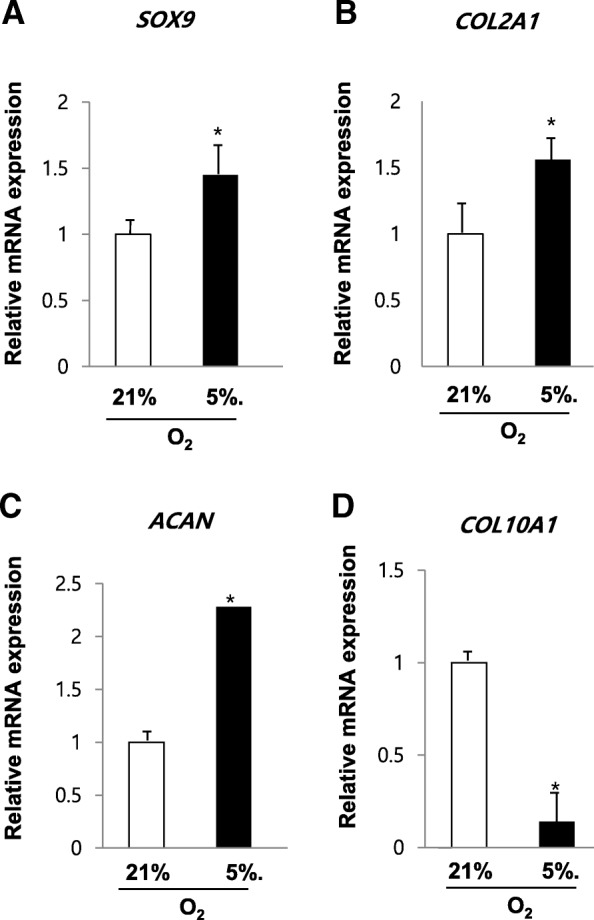
Hypoxia induces chondrogenesis-related gene expression in SDSCs. SDSCs were isolated, and subsequently differentiated under normoxia or hypoxia for 21 days in chondrogenic medium. Quantitative RT-PCR analysis of gene expression with hypoxia- and normoxia-cultured SDSCs. (**a**) *SOX9*, (**b**) *COL2A1*, (**c**) *ACAN*, and (**d**) *COL10A1* mRNA level

To determine the effects of hypoxic culture on SDSCs chondrogenesis, we validated the mRNA levels of the transcription factor SOX9, types II collagen, aggrecan, and types X collagen in chondrogenically differentiating SDSCs under normoxic and hypoxic conditions by quantitative RT-PCR. In the chondrogenic cultures under hypoxia condition, the gene expression of *COL2A1*, *ACAN*, and the transcription factor *SOX9* was greatly increased in comparison with nomoxia condition. The expression of *SOX9*, *COL2A1*, and *ACAN* at lowered oxygen tension was increased 1.4-fold, 1.6-fold, and 2.3-fold, respectively (*p* < 0.05; Fig. 3). In contrast, the hypertrophic cartilage-enriched gene transcripts of *COL10A1* mRNA levels were strongly down-regulated under hypoxia conditions compared with nomorxia conditions (*p* < 0.05).

### Hypoxic culture enhances chondrogenesis in SDSCs

**Fig. 4:**
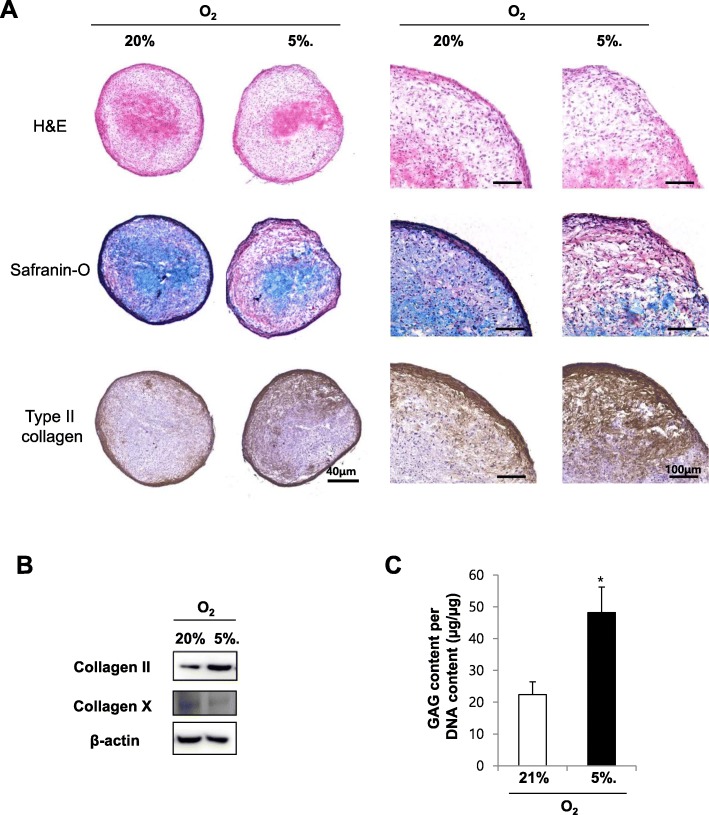
Hypoxic culture enhances chondrogenesis in SDSCs. Histological analysis of chondrogenic cultures of SDSCs. SDSCs (aliquots of 2.0 × 10^5^) were pelleted and induced in chondrogenic differentiation medium under normoxic (21% O_2_) and hypoxic (5% O_2_) conditions for 21 days. (**a**) H&E staining, Safranin-O staining for proteoglycan, and immunohistochemical staining for collagen type II after chondrogenic differentiation for 21 days under normoxic and hypoxic culture conditions. Right panel is high magnification images (**b**) Western blot of collagen II and X (**c**) Quantification of glycosaminoglycan (GAG) contents

Pellets of SDSCs were differentiated into the chondrogenic lineage for up to 21 days in the presence of TGF-β in either normoxic or hypoxic conditions. Safranin-O staining was used to assess ECM proteoglycan content within SDSCs pellet after 21 days of chondrogenic differentiation. Histological sections stained with Safranin-O demonstrated that hypoxic conditions had increased proteoglycan synthesis compared to normoxic culture on day 21 of chondrogenesis (Fig. 4a). Immunohistochemistry verified the presence of collagen II within pellet that was exposed to hypoxia during differentiation. Immunohistochemistry for collagen II demonstrated that hypoxic culture of SDSCs increased type II collagen expression compared to normoxic culture (Fig. 4a). To confirm the result from pellet culture, we examined protein level of collagen types II and X by western blot. Under hypoxia condition, the protein expression of collagen types II was greatly increased in comparison with nomoxia condition. In contrast, the protein level of collagen types X was down-regulated under hypoxia conditions compared with nomorxia conditions (Fig. 4b). We also confirmed a biochemical quantification of glycosaminoglycan (GAG) contents from pellet cultured in normoxic or hypoxic conditions. GAG deposition significantly higher in hypoxia compared with normoxia at 21 days of differentiation (Fig. 4c). Together, these data suggested that hypoxic conditions increased chondrogenesis of SDSCs.

## Discussion

Because articular cartilage has a poor self-healing capacity, it is difficult to properly manage patients who have cartilage injuries [19]. Since MSCs are a source of cells for developing novel engineered tissue constructs for treatment, of musculoskeletal diseases, such as cartilage defects, their manipulation in vitro has received significant attention in the past decade.

All of MSCs share the characteristics of self-renewal and differentiation into multi-lineage cell types such as osteocytes, chondrocytes and adipocytes [20–22]. Previous studies have indicated that SDSCs are a promising cell source for the repair and regeneration of cartilage [23]. The use of SDSCs is not prevented by the limited availability of healthy articular cartilage or an intrinsic tendency of the cells to lose their phenotype during expansion, and the use of SDSCs avoids the morbidity caused by damage to the donor-site articular surface. Synovium may serve as a source of MSCs that are mobilized following injury, and the MSCs migrate to the wound site where, they participate in the repair response [24]. After partial chondral defects were created in the rabbit articular cartilage, a continuous layer of MSCs extending from the synovium was found to contribute to cartilage regeneration. We have previously shown that the SDSCs can be expanded in culture and differentiated into the desired lineage with the application of specific growth factors [25]. To regenerate damaged articular cartilage, it is necessary to identify an appropriate cell source that is easily accessible, can be expanded to large numbers, and has chondrogenic potential. Therefore, we believe that SDSCs are the most clinically promising source of stem cells to develop new strategies for cartilage regeneration. SDSCs have recently been explored as an alternative cell source for cartilage regeneration and repair because of their chondrogenic potential and their ease of isolation from sources, such as joints, without damage to the native cartilage tissue. Prior to the application of SDSCs, in vitro expansion and appropriate chondrogenic induction methods are indispensable.

Chondrogenic differentiation of MSCs may be induced by specific cytokines and growth factors, biophysical stimulation, and provision of a suitable 3-dimensional environment [6–8]. Although the factors that influence optimal MSC chondrogenesis remain to be fully elucidated, one of them, oxygen tension seems to be an important regulatory factor in the proliferation, differentiation and matrix production of chondrocytes [26, 27, 29]. The hypoxic condition has proven to be beneficial to some kinds of MSC. Low oxygen tensions enhanced expansion potential of human umbilical cord -derived stem cells [12]. Hypoxia could promote the growth of NSCs and maintain its survival in vitro [26]. The chondrogenic marker genes and transcription factors, including *SOX5*, *SOX6*, *SOX9*, *type II collagen*, *type IX collagen*, *type XI collagen*, *aggreca (ACAN)* and *versican*, were all significantly enhanced under hypoxia (5% O_2_) compared with those under normoxia during chondrogenic differentiation of human MSCs (hMSCs) derived from infrapatellar fat pad [28]. Chondrogenic differentiation of hBM-MSCs was greatly enhanced under hypoxia (5% O_2_) [7, 9, 10]. Moreover, articular hyaline cartilage is avascular in nature, having no nerves or blood supply [13, 14]; therefore oxygen could only diffuse from the synovial fluid. Thus, there appears to be a gradient of decreasing oxygen tension from the surface of the articular cartilage to the subchondral bone, and the physiological oxygen tension of articular chondrocytes is 5–10% at the surface and possibly, 1% in the deepest layer. The chondrocytes of articular cartilage can survive with maintained phenotype under low oxygen tension.

These evidences have suggested that low oxygen tension seems to be an important regulatory factor in the proliferation and chondrogenic differentiation in SDSCs. But, the effect of low oxygen tension on the proliferation and chondrogenic differentiation in SDSCs has not characterized. Our data showed that low oxygen condition significantly increased proliferation and colony-forming characteristics of SDSCs compared to that of SDSCs under normoxic culture. The mechanism of these responses might be primarily involved in the hypoxic inducible factor-1 (HIF-1) and/or PI3K/AKT/Foxo signal pathway [26, 28, 34]. The mRNA expression of types II collagen, aggrecan, and the transcription factor SOX9 was increased under hypoxia condition. Histological sections stained with Safranin-O demonstrated that hypoxic conditions had increased proteoglycan synthesis. Immunohistochemistry data demonstrated that hypoxic culture of SDSCs increased type II collagen expression. We also confirmed a biochemical quantification of glycosaminoglycan (GAG) contents from pellet cultured in normoxic or hypoxic conditions. GAG deposition significantly higher in hypoxia compared with normoxia at 21 days of differentiation. Although our data suggested that chondrogenesis and proliferation of SDSCs can be enhanced under hypoxic conditions, the underlying mechanisms that hypoxic conditions mediate still remain unclear. The response by cells to hypoxia is complex and is mediated by several genes. HIF1α may be candidate of the major regulators of hypoxic response in SDSCs. Targets of its molecular signaling are reported to include a cluster of hydroxylases that are crucial for collagen fibre formation such as prolyl 4-hydroxylase and procollagen lysyl-hydroxylase [30–32]. Through these actions, HIF1α may affect the rate of synthesis of procollagen chains in vivo and in vitro. Thus, further studies should be performed to obtain more information on how low oxygen tension modulates chondrogenic effects in SDSCs.

## Conclusion

Oxygen tension seems to be an important regulatory factor in the proliferation, differentiation and matrix production of chondrocytes. But, the effect of low oxygen tension on the proliferation and chondrogenic differentiation in SDSCs has not characterized. In this study, we investigated the effects of hypoxia on chondrogenesis in SDSCs. Our data indicate that low oxygen condition significantly increased proliferation and chondrogensis of SDSCs compared to that of SDSCs under normoxic culture. These findings suggest that hypoxia condition has an important role in regulating the synthesis ECM matrix by SDSCs as they undergo chondrogenesis. This has important implications for cartilage tissue engineering applications of SDSCs.
