# Correction to: Hypoxic condition enhances chondrogenesis in synovium-derived mesenchymal stem cells

**DOI:** 10.1186/s40824-019-0158-x

**Published:** 2019-03-04

**Authors:** Hyun Cheol Bae, Hee Jung Park, Sun Young Wang, Ha Ru Yang, Myung Chul Lee, Hyuk-Soo Han

**Affiliations:** Department of Orthopedic Surgery, Seoul National University Hospital, Yongondong, Chongnogu, Seoul, 110-744 Republic of Korea

**Correction to: Biomater Res**

**https://doi.org/10.1186/s40824-018-0134-x**

The original article [1] contains errors in the Acknowledgements and Funding sections of the Declarations. The two corrected sections are as follows:

**Acknowledgements**

Not applicable.

**Funding**

This study was funded by Hanmi Pharm. The funding source had no role in the study design, collection, analysis or interpretation of the data, writing of the manuscript, or in the decision to submit the manuscript for publication.
